# Smell disorders in COVID-19 patients: role of olfactory training: A protocol for systematic review and meta-analysis

**DOI:** 10.1097/MD.0000000000024862

**Published:** 2021-02-26

**Authors:** Yu Zhang, Tao Mei, Ying Chen, Lina Wang, Lulian Jiang, Ke Liu, Liping Zhao, Ziyu Luo, Wenxin Chi, Xiangyu Zhu

**Affiliations:** a School of Acupuncture-Moxibustion and Tuina, Beijing University of Chinese Medicine; b China Institute of Sport and Health Science, Beijing Sport University; c Dongzhimen Hospital; d Laboratory of Statistics and Measurement, Beijing Sport University, Beijing, China.

**Keywords:** COVID-19, meta-analysis, olfactory training, smell disorders, systematic review

## Abstract

**Background:**

As the coronavirus disease 2019 (COVID-19) spread around the world, a surge of evidence suggests that smell disorders are common symptoms in COVID-19 infection. This dysfunction may cause loss of appetite, malnutrition, poisoning, and depression. Obviously, the impairment has a strong impact on the quality of life. Therefore, there is an urgent need to identify effective treatments. Various therapies have been studied to treat smell disorders after infection, and olfactory training (OT) is considered a promising treatment option. Assessing the effectiveness and safety of olfactory training for COVID-19 patients with smell disorders is the main purpose of this systematic review protocol.

**Methods:**

PubMed, EMBASE, MEDLINE, the Cochrane Library, Chinese National Knowledge Infrastructure, Chinese Biomedical Literature Database, Wanfang Database, ClinicalTrials.gov trials registry, and Chinese Clinical Trial Registry will be searched from January 2019 to January 2021. A combination of subject words and free text words will be applied in the searches. The language is limited to Chinese and English. The complete process will include study selection, data extraction, risk of bias assessment, and meta-analyses. Endnote X9.3 will be used to manage data screening. The statistical analysis will be completed by Review Manager V.5.3 (Cochrane Collaboration) or Stata V.16.0 software.

**Results:**

This proposed study will assess the effectiveness and safety of OT for COVID-19 patients with smell disorders.

**Conclusion:**

The conclusion of this study will provide evidence to prove the effectiveness and safety of olfactory training for COVID-19 patients with smell disorders.

**Ethics and dissemination:**

This protocol will not evaluate individual patient information or infringe patient rights and therefore does not require ethical approval.

**Registration:**

PEROSPERO CRD42020218009.

## 1 Introduction

The coronavirus disease 2019 (COVID-19), an acute respiratory disease, caused by a novel coronavirus (SARS-CoV-2, previously known as 2019-nCoV), has rapidly spread across the world and reached pandemic level by March 2020.^[1,2]^ Its extraordinary human to human transmission efficiency leading to irrepressibly increasing global incidents on such an alarming rate exhibited high potential for a pandemic.^[3,4]^

The symptoms of COVID-19 found in the early stage of epidemic are fever, cough, fatigue, slight dyspnoea, sore throat, headache, conjunctivitis, and gastrointestinal issues.^[5]^ However, as the investigation continues, smell disorders as new symptoms have recently appeared in several publications, bringing about widespread attention, from which an exciting direction of research about COVID-19 emerged.^[6]^ Smell disorders have been increasingly reported in individuals infected with SARS-CoV-2.^[7]^ A previously published systematic review suggests a prevalence of self-reported smell disorders in 50% of patients with COVID-19.^[8,9]^ As of December 20, there have been over 75 million cases since the start of the pandemic.^[10]^ According to the ratio, there have been more than 35 million of them suffering from smell disorders. For the high prevalence among COVID-19, smell disorders have been included in the official lists of symptoms worldwide.^[11]^

Smell disorders have a strong impact on the quality of life, these impairments affect the ability to sense odors in foods and the environment, it may lead to malnutrition, weight loss, food poisoning, depression, and exposure to dangerous chemicals. Smell disorders have been also associated with increased mortality.^[12]^ High prevalence and severe effects make it an urgent need to seek for effective treatments.

So far, studies have suggested different probable mechanisms for the development of smell disorders in COVID-19, including olfactory cleft syndrome with mucosal obstruction, post-viral anosmia syndrome, cytokine storm, direct damage of olfactory sensory neurons, and impairment of the olfactory perception center in the brain.^[13,14]^ The uncertainty of pathogenesis makes it difficult to determine the treatment. However, since Post-viral olfactory dysfunction (PVOD) is the most common cause of smell disorders and since coronaviruses are one of many pathogens, it is reasonable to deem the smell disorders in COVID-19 as a classification of PVOD.^[9,15,16]^ Thus, evaluating the effective treatment for PVOD is of great significance. A wide range of treatment modalities including olfactory training (OT), corticosteroid, theophylline, budesonide (nasal irrigation), intranasal calcium buffers, and antibiotics have been attempted to treat PVOD.^[17–19]^ Among them, OT is the only evidence-based therapeutic option for PVOD.^[15]^ Its application in PVOD shows promising results.^[20]^ OT has been demonstrated that it can efficiently cure smell disorders induced by upper respiratory tract infection, and in particular, significantly improve the odor discrimination ability and odor identification ability.^[21]^ Meanwhile, it is reported that OT can also decrease depressive symptoms in patients with smell disorders.^[22]^ For its efficacy, OT has been widely used by clinic.^[23]^

As for the cure rate of smell disorders caused by COVID-19, research suggests OT can achieve better therapeutic effects at the early stage.^[24]^ Research from Dr Borsetto D and Dr Hopkins C shows a high rate of early recovery, at 4 to 6 weeks after onset, approximately 90% patients have experienced recovery.^[8,9]^ Dr Yan XG's study also suggests in patients with less than a year of smell disorders, the olfactory function improved obviously.^[24]^ In addition, long-term OT seems to be associated with better results in patients with smell disorders than a short-term scheme.^[25]^ So OT is strongly recommended when smell does not come back after 1 month but can be started earlier.^[12]^

Solid evidence for the efficiency of OT in PVOD and the belonging of the smell disorders in COVID-19 to PVOD show great possibility that OT can improve smell disorders in COVID-19. Therefore, we will investigate the effectiveness and safety of OT for COVID-19 patients with smell disorders in a systematic review and meta-analysis.

## 2 Methods

### 2.1 Registration

The study protocol has been registered on international prospective register of systematic review (PROSPERO registration number: CRD42020218009). The procedure of this protocol will be conducted according to the Preferred Reporting Item for Systematic Review and Meta-analysis Protocols guidance.^[26]^

### 2.2 Inclusion and exclusion criteria

#### 2.2.1 Type of study

Randomized controlled trials about Olfactory Training for smell disorders caused by COVID-19 will be included. Studies will be excluded if they are:

1. Nonrandomized controlled trials, literature review, case-control trials, and animal research literature
2. Studies with repeated publication, unclear outcome measures, and obvious data errors
3. Studies with fewer than 10 samples

#### 2.2.2 Type of participant

Subjects with documented COVID-19 with Olfaction Disorders of 4 weeks duration or longer. Inclusion criteria:

1. Adult women and men.
2. Positive laboratory finding for SARS-CoV-2.
3. In convalescence from their COVID-19 illness.
4. Subjective complaints of reduced olfaction after COVID-19 infection of greater than 4 weeks duration.
5. Ability to read, write, and understand English.

Exclusion criteria

1. History of olfactory disorder prior to COVID-19 infection.
2. History of nasal cavity polyps.
3. Dependence on prolonged corticosteroid therapy for comorbid conditions, such as asthma and chronic obstructive pulmonary disease.
4. History of cerebrospinal fluid leak.
5. History of allergy to budesonide or other topical steroids.

#### 2.2.3 Type of interventions

Interventions are OT and periodical assessment to track progress of training. Multiple control measures will be included, such as blank, placebo, and drug therapy. Any comparisons between a combined therapy of OT and other interventions with a therapy of solely using other interventions are also included.

#### 2.2.4 Type of outcome measures

Outcome indicators include effectiveness indicators and safety indicators. Effectiveness indicators include primary outcome indicators and secondary outcome indicators. The primary outcome indicators are the scores of olfactory function using validated tools such as Sniffin’ Sticks test, University of Pennsylvania Smell Identification Test, Questionnaire of Olfactory Disorders-Negative Statements, Global Rating of Smell, Global Rating of Smell Change. The secondary outcome indicators are the scores of quality of life using tools like SF-36, NHP, World Health Organization Quality of life scale (whoqol-1000), and the quality of life index. Safety is referred to the incidence of adverse events.

### 2.3 Search strategy

PubMed, EMBASE, MEDLINE, the Cochrane Library, Chinese National Knowledge Infrastructure, Chinese Biomedical Literature Database, Wanfang Database, ClinicalTrials.gov trials registry, and Chinese Clinical Trial Registry will be searched from January 2019 to January 2021. A combination of subject words and free text words will be applied in the searches. The language is limited to Chinese and English. The search terms are shown in Table 1.

**Table 1:** Search strategy of PubMed.

Search	Query
#1	smell disorders[MeSH Terms]
#2	“smell disorder”[Title/Abstract] OR “olfaction disorder”[Title/Abstract] OR “dysosmias”[Title/Abstract] OR “dysosmia”[Title/Abstract] OR “cacosmias”[Title/Abstract] OR “cacosmia”[Title/Abstract] OR “paraosmia”[Title/Abstract] OR “anosmia”[Title/Abstract] OR “hyposmia”[Title/Abstract] OR “olfactory hallucination”[Title/Abstract]
#3	#1 or #2
#4	COVID-19[MeSH Terms]
#5	(((((((((((((coronavirus disease 2019[Title/Abstract]) OR (2019-nCoV disease[Title/Abstract])) OR (2019-nCoV infection[Title/Abstract])) OR (COVID 19[Title/Abstract])) OR (COVID 2019[Title/Abstract])) OR (COVID19[Title/Abstract])) OR (nCoV 2019 disease[Title/Abstract])) OR (nCoV 2019 infection[Title/Abstract])) OR (novel coronavirus 2019 disease[Title/Abstract])) OR (novel coronavirus 2019 infection[Title/Abstract])) OR (novel coronavirus disease 2019[Title/Abstract])) OR (novel coronavirus infection 2019[Title/Abstract])) OR (Wuhan coronavirus disease[Title/Abstract])) OR (Wuhan coronavirus infection[Title/Abstract])
#6	#4 or #5
#7	olfactory training[MeSH Terms]
#8	“randomized controlled trial”[Publication Type] OR “randomized”[Title/Abstract] OR “placebo”[Title/Abstract]
#9	#3 and #6 and #7 and #8

### 2.4 Study selection

**Figure 1:**
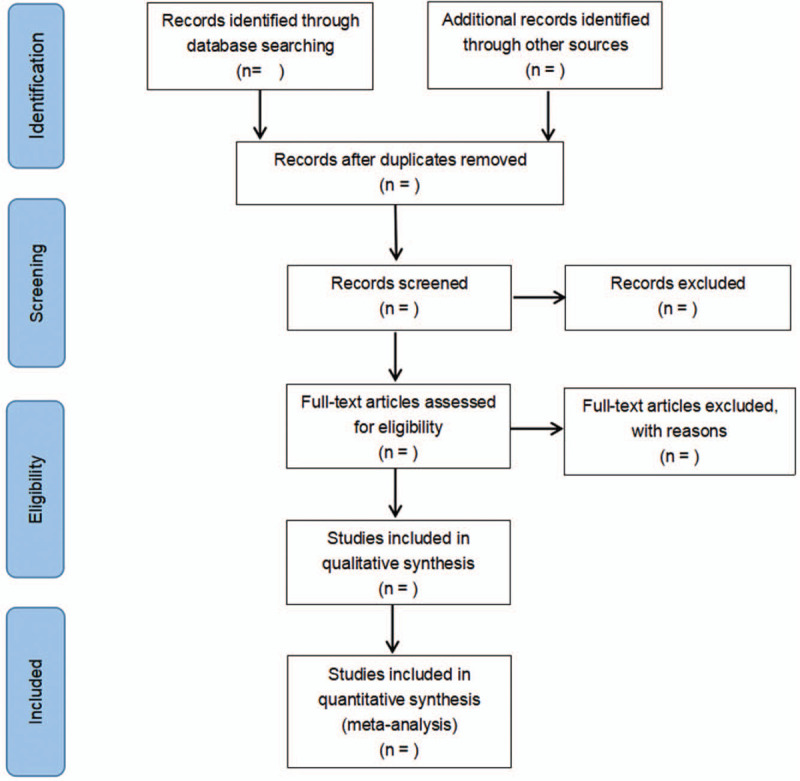
PRISMA flow chart presenting details of the selection process.

The literature will be retrieved according to the retrieval strategy, then imported them into the literature management software. Endnote version 9.3 (The Thomson Corporation Corp, Stanford, CT) will be used to manage data screening. The research on duplicate titles was deleted, and obviously irrelevant literature was excluded by reading titles and abstracts. The above steps were performed independently by 2 researchers. Any disagreements will be resolved by discussion with third researchers. The researchers will record all studies that do not meet the inclusion criteria and provide the rationale for their exclusion. Details of the selection process will be presented in the PRISMA flow chart (Fig. 1).

### 2.5 Data extraction

The studies retrieved during the searches will be filtered, the data extracted, and a database constructed. The literature will be retrieved according to the search strategy, then imported into the literature management software. EndNote X9.3, which will be used to manage the data screening. Duplicate titles will be deleted, and obviously irrelevant literature excluded based on an assessment of the titles and abstracts. The remaining literature will then be downloaded and read, and that which does not conform to the eligibility criteria will be excluded. Data from the selected studies will then be extracted, and will include general information, reference information (name of the leading author and year of publication, and study design), participant characteristics, intervention details, methods, controls, training frequency and duration, the outcomes measured, the results, and any adverse reactions. The above steps will be performed independently by 2 researchers, and any disagreements will be resolved by discussions with a third reviewer.

### 2.6 Risk of bias assessment

Two authors will assess methodological quality of included studies separately by the Cochrane collaboration's risk of bias tool.

We will consider the following:

1. random sequence generation (selection bias)
2. allocation concealment (selection bias)
3. blinding of participants and personnel (performance bias)
4. blinding of outcome assessment (detection bias)
5. incomplete outcome data (attrition bias)
6. selective reporting (reporting bias)
7. other sources of bias (other bias).

The bias risk in each aspect will be assessed and divided into 3 levels: low risk, high risk, and unclear risk. The 2 authors will resolve any disagreements through discussion, and will reach consensus through a third reviewer.

### 2.7 Statistical analysis

#### 2.7.1 Strategy for data synthesis

Review Manager V.5.3 (Cochrane Collaboration) or Stata V.16.0 software will be used to conduct the meta-analysis. The groups included in the synthesis must meet our inclusion criteria. Heterogeneity will be assessed using the Q test (with P 0.1 being considered to represent significant statistical heterogeneity), and the I^2^ statistic (with values of I^2^ 50% considered to be indicative of substantial heterogeneity). If necessary, meta-analysis, subgroup, and sensitivity analyses will also be performed to analyze the source of any heterogeneity. The data synthesis will be conducted using a random-effects or fixed-effect model. We will clearly describe which studies have been included, and how they have been synthesized as described. We will also be transparent about the metrics being used, and endeavor to include confidence intervals with our synthesis findings.

#### 2.7.2 Analysis of subgroups or subsets

If there is a significant level of heterogeneity in the included studied, subgroup analyses will be performed according to gender and age. If necessary, meta-regression and sensitivity analyses will also be performed to analyze the source of any heterogeneity.

#### 2.7.3 Sensitivity analysis

Different levels of the methodological quality of trails may tend to affect the overall effects. If the Q test and the I^2^ statistic show significant statistical heterogeneity, sensitivity analyses we will conduct sensitivity analysis. Sensitivity analysis is conducted by excluding studies one by one, so that we can determine the source of heterogeneity.

#### 2.7.4 Publication bias

The publication bias will be evaluated by funnel plots by determining whether there are 10 or more studies with the same outcome. In the case of asymmetric funnel plot, subgroup analysis or sensitivity analysis will be performed to investigate possible causes.

#### 2.7.5 Quality of evidence

We will use the Grading of Recommendations Assessment, Development, and Evaluation guidelines for the assessment of the strength of evidence for each outcome. The result will be categorized as high, moderate, low, and very low certainty of evidence.

### 2.8 Ethics and dissemination

This systematic review will not require ethical approval because there are no data used in our study that are linked to individual patient data.

## 3 Results

This proposed study will evaluate the effectiveness and safety of OT for patients with COVID-19-related smell disorders.

## 4 Discussion

The recent COVID-19 outbreak around the world has had an enormous impact on the global health burden, resulting in overwhelming number of reported deaths, and has had severe socio-economic consequences.^[27,28]^ While actively treating patients to reduce mortality and developing vaccines to reduce infection rates, should also pay attention to improve the quality of life of survivors. Smell disorders occur in COVID-19 with a high prevalence.^[29]^ This inability has a significant negative impact on quality of life, causing a series of adverse consequences.^[12,30]^ Given serious adverse effects of smell disorders and the fast growing number of patients, it is momentous to find out a valid treatment.

Because the pathogenesis is still under study, it is tough to determine the accurate treatment therefrom. However, researches have shown that PVOD has become especially relevant with the onset of the COVID-19 pandemic.^[31,32]^ Since coronavirus is one of the triggers of PVOD, the effective treatment of PVOD is of great reference value for studying the treatment of smell disorders in COVID-19.^[9,15]^ Present study shows that OT, as an emerging nonpharmacologic therapy option, has shown promise in the treatment of PVOD.^[33,34]^ An increasing body of preclinical and clinical evidence supported the beneficial effects of OT in smell disorders.^[33–41]^ Compared with other treatment modalities, OT is an effective treatment strategy with low cost and negligible adverse effects.^[42]^ Except for its own advantages, lacking of specific pharmacologic therapies for PVOD further supports the contemporary interest in OT as a treatment option for patients with PVOD.^[43]^

As the above advantages, OT is recommended for patients with COVID-19-related smell disorders.^[12]^ At present, there are no systematic reviews of the effects of OT for the patients with smell disorders caused by COVID-19. It is hoped that this meta-analysis can provide convincing scientific basis and guide clinical practice.

## 5 Conclusion

The conclusion of this study will provide evidence to prove the effectiveness and safety of OT on patients with COVID-19-related smell disorders.

## Acknowledgments

This work was supported by the School of Acupuncture, Moxibustion and Tuina, Beijing University of Chinese Medicine.

## Author contributions

XYZ and YZ participated in the design of the study, XYZ participated in the literatures collection and analysis; YC and ZYL participated in data collection; LNW participated in Literature data extraction and quality assessment; TM and WXC participated in the design of the study and contributed to literatures collection; LLJ, LPZ, and KL participated in the revisions of the study; All authors contributed to the manuscript writing. All authors have read and approved the final version of the manuscript, and agree with the order of presentation of the authors.

**Conceptualization:** Yu Zhang, Xiangyu Zhu.

**Data curation:** Lina Wang.

**Formal analysis:** Tao Mei.

**Methodology:** Ziyu Luo.

**Resources:** Ying Chen.

**Software:** Ke Liu.

**Supervision:** Lulian Jiang.

**Validation:** Lina Wang, Wenxin Chi.

**Visualization:** Liping Zhao.

**Writing – original draft:** Xiangyu Zhu.

**Writing – review & editing:** Xiangyu Zhu.
